# Microbes and Internal Medication

**Published:** 1897-10-23

**Authors:** 


### MICROBES AND INTERNAL MEDICATION.

The experience which has been accumulated in regard to the action of vaccines and anti-toxins has been taken by some as encouraging the view that some protective drug may be discoverable, and we are not going to suggest that such a thing is absolutely outside the range of what is possible. It is only right, however, to emphasise the fact that the substances which have proved of undoubted efficacy in this direction have so far been substances which have been produced in the laboratory of nature, not in that of the chemist, and that most attempts to prevent microbial diseases by soaking man's tissues with crude chemical, and especially mineral, products have proved unavailing. Some interesting observations in regard to this point have recently been made by M. Albert Robin, who has related cases in which patients fully charged in the one case with lead, and the other with mercury, were attacked by infectious disorders, notwithstanding the assumed antiseptic influence of such a saturation of the system with metallic salts.

The fact has to be recognised that every organism resists the intrusion of other organisms in its own way; by dint, that is, of some property peculiar to living tissues, and that, whether we dub that property "vitality," or "power of resistance," or "immunity," or what not, it is only by stimulating this normal activity of tissue-resistance that we can hope to influence for good the progress of an infective disease.

